# Accuracy of a *Plasmodium falciparum* specific histidine-rich protein 2 rapid diagnostic test in the context of the presence of non-malaria fevers, prior anti-malarial use and seasonal malaria transmission

**DOI:** 10.1186/s12936-017-1941-6

**Published:** 2017-07-20

**Authors:** Francois Kiemde, Massa dit Achille Bonko, Marc Christian Tahita, Palpouguini Lompo, Toussaint Rouamba, Halidou Tinto, Michael Boele van Hensbroek, Petra F. Mens, Henk D. F. H. Schallig

**Affiliations:** 1 Institut de Recherche en Science de la Sante-Unite de Recherche Clinique de Nanoro, Nanoro, Burkina Faso; 2 Global Child Health Group, Academic Medical Centre, University of Amsterdam, Amsterdam, The Netherlands; 3 Department of Medical Microbiology, Academic Medical Centre, Parasitology Unit, Amsterdam, The Netherlands

**Keywords:** RDT-*Pf*HRP2, Diagnosis, Malaria, Fever, Sensitivity, Specificity, Accuracy

## Abstract

**Background:**

It remains challenging to distinguish malaria from other fever causing infections, as a positive rapid diagnostic test does not always signify a true active malaria infection. This study was designed to determine the influence of other causes of fever, prior anti-malarial treatment, and a possible seasonality of the performance of a *Pf*HRP2 RDT for the diagnosis of malaria in children under-5 years of age living in a malaria endemic area.

**Methods:**

A prospective etiology study was conducted in 2015 among febrile children under 5 years of age in Burkina Faso. In order to assess the influence of other febrile illnesses, prior treatment and seasonality on the performance of a *Pf*HRP2 RDT in diagnosing malaria, the RDT results were compared with the gold standard (expert microscopic diagnosis of *Plasmodium falciparum*) and test results were analysed by assuming that prior anti-malarial use and bacterial/viral infection status would have been known prior to testing. To assess bacterial and viral infection status blood, urine and stool samples were analysed.

**Results:**

In total 683 blood samples were analysed with microscopy and RDT-*Pf*HRP2. *Plasmodium falciparum* malaria was diagnosed in 49.8% (340/683) by microscopy compared to 69.5% (475/683) by RDT-*Pf*HRP2. The RDT-*Pf*HRP2 reported 29.7% (141/475) false positive results and 1.8% (6/340) false negative cases. The RDT-*Pf*HRP2 had a high sensitivity (98.2%) and negative predictive value (97.1%), but a low specificity (58.9%) and positive predictive value (70.3%). Almost 50% of the alternative cause of fever were diagnosed by laboratory testing in the RDT false positive malaria group.

**Conclusions:**

The use of a malaria RDT-*Pf*HRP2 in a malaria endemic area may cause misdiagnosis of the actual cause of fever due to false positive test results. The development of a practical diagnostic tool to screen for other causes of fever in malaria endemic areas is required to save lives.

## Background

With the recommendation of the World Health Organization (WHO) to confirm a malaria infection in a febrile child before commencing treatment, malaria rapid diagnostic tests (RDTs) have become indispensable in the screening of malaria-suspected fever cases [1]. The main goal of this positive development is to substantially reduce unnecessary prescription of anti-malarials and hence decrease inappropriate treatment [2, 3]. However, persisting antigen after adequate treatment or spontaneous remission, which can be detected by the RDT employed, might jeopardize this aim [4]. This is most evident in the case of the diagnostic target antigen histidine-rich protein-2 (*Pf*HRP2), which is specific to *Plasmodium falciparum*, and that can persist in the blood for weeks after treatment [5–7]. This antigen persistence could increase the rate of false positive test results, leading to a wrong diagnosis in settings where other diagnostic tools are unavailable. This could in particular be the case when the fever persists or relapses after malaria treatment. If malaria is successfully treated the *Pf*HRP2-based RDT (RDT-*Pf*HRP2) will remain positive, suggesting a malaria infection, whereas the actual cause of fever is due to another infection [2, 3, 8]. This phenomenon of false positive results after successful treatment can result in an overestimation of malaria positive cases and lead to misdiagnosis of the true cause of fever in children. Recent studies have reported a decreasing specificity and positive predictive value of the RDT-*Pf*HRP2 up to 3–4 weeks after successful malaria treatment [2, 3, 9, 10]. This period of antigen persistence leads to a diagnostic gap in which HRP-2 based tests cannot be used. Although RDT-*Pf*HRP2 allows to treat a large part of children who actually have malaria, the children with false positive results could be suffering from alternative causes of fever like bacterial or viral infections [9].

Up to date several studies have assessed the performance of malaria RDT-*Pf*HRP2 in different malaria transmission areas under controlled conditions. For example, Grandesso et al. [5] showed that the sensitivity of two different HRP-2 based tests in a low or high transmission area ranged from 98.4 to 99.2%, with no significant difference between tests and settings. However, the specificity of the HRP-2 based tests was much lower in high transmission settings (79.7–80.7%) compared to the low transmission settings (98.8–98.8%). In general, the specificity of HRP-2 based RDTs seems to decrease with an increase in *P. falciparum* malaria [11]. This specificity issue of the HRP2 RDT may thus affect its usefulness in health care practice. However, limited data are available on the influence of other causes of fever on the performance of this RDT in rural areas where mainly febrile children attending the health facilities are being screened with malaria RDTs to determine the cause of their fever. If the performance of the RDT-*Pf*HRP2 is affected by other infections in malaria endemic areas, the end result might be that other potential causes of fever are being ignored, and that the fever is still being treated as if it is malaria.

In Burkina Faso, like many other malaria endemic areas, the introduction of malaria RDT-*Pf*HRP2 significantly contributed to reducing anti-malarial prescriptions, mainly in children [12]. However, as an unwanted effect it could increase the untargeted use of antibiotics to treat fever [13]. Previous studies in Burkina Faso have reported on the performance of malaria RDT-*Pf*HRP2 tests in relation to the accuracy of a RDT for the diagnosis of both malaria and malaria-attributable fever or to other infections [14, 15]. However, there are no studies from Burkina Faso assessing the number of wrongly diagnosed cases with other febrile diseases when only the results of the RDTs are used for patient management. Therefore, the present study was designed to determine the influence of other causes of fever and previous malaria infections on the performance of RDT-*Pf*HRP2 for the diagnosis of malaria in children under-5 years living in a malaria endemic area.

## Methods

### Study site

The study was conducted in the health district of Nanoro, located in central-west part of Burkina Faso. Nanoro is around 100 km from Ouagadougou, the capital of the country. The data were collected in four peripheral health facilities of the health district of Nanoro (i.e. Nanoro, Godo, Nazoanga and Seguedin) and the referral hospital Saint Camille of Nanoro. These peripheral health facilities have been chosen as study locations because of their accessibility and close distance (the most remote is 20 km) from the central laboratory. CMA (Centre Medical avec Antenne Churigicale) Saint Camille of Nanoro was included to increase inclusion of severe cases that were referred from peripheral health facilities. The district hospital CMA Saint Camille de Nanoro is the reference hospital of the health district of Nanoro with trained medical staff and equipped laboratory facilities for the management of difficult medical cases that are referred from the peripheral health facilities. The peripheral health facilities are the first point of medical contact within the community for the management of less complicated medical cases by Community Health Workers based on guideline of diseases management [16]. Malaria is the first cause of consultation in children under-5 years of age in this region and predominately occurs during the rainy season July–November.

### Study design

This study has been conducted as part of a larger project that aims to improve the diagnosis and management of non-malaria fevers in children under-5 years of age in Nanoro, Burkina Faso. Briefly, all children under-5 years of age documented with axillary temperature ≥37.5 °C presenting at the participating health facilities were invited to participate in the study. Written informed consent was obtained from parent/guardian before any data collection. All participants were tested systematically for malaria infection with RDT and managed according to the test result as per National Malaria Control Programme (NMCP) guidelines. In addition, expert malaria microscopy was performed for the purpose of studying the fever aetiology and the accuracy of the RDT-*Pf*HRP2 employed. Furthermore, clinical specimens like blood and urine for bacterial cultures, and stool for rotavirus and adenovirus tests were also collected systematically and analysed at the microbiology laboratory of the Clinical Research Unit of Nanoro (CRUN). This study was approved by the National Ethical Committee in Health Research, Burkina Faso (Deliberation No. 2014-11-130).

### Laboratory procedure

#### Malaria rapid diagnostic test

The malaria Rapid Diagnostic Test recommended by the Burkinabe NMCP is the SD Ag Bioline *Pf* (Standard Diagnostics, Hagal-Dong, Korea) detecting *Pf*HRP2 and this test was performed by trained study nurses at the health facilities or the district hospital. The nurses recorded malaria RDT results as positive or negative on study case record forms. During the last WHO-FIND testing round 6 the specificity and sensitivity for this particular test was reported to be high (>95%) [17].

#### Microscopy

Malaria diagnosis by microscopy was performed by expert laboratory technicians at CRUN. These expert microscopists are frequently submitted to an external quality control programme and only certified microscopists are allowed to read the slides. The LoD of this expert microscopy 10 parasites/µl. Thick and thin blood smears were prepared (in duplicate) from blood collected in the Ethylene Diamine Tetra Acetic acid (EDTA) tubes. Thin films were fixed with methanol and blood slides were stained with 3% Giemsa solution (pH 7.2) for identification and quantification of asexual *P. falciparum* and others *Plasmodium* species. Parasites densities were determined by counting the number of asexual parasites per 200 white blood cells, and calculating per μl of blood by assuming the number of white blood cells to be at 8000/μl. Thick blood smears were considered negative when the examination of 100 fields per thick film did not reveal the presence of any asexual parasites. Each blood slide was read by two independent expert readers, and in case of discordance (positive *vs* negative, different in *Plasmodium* species, difference in parasite density >Log10 or ratio >2 in case of parasite density ≤400 or >400/µl, respectively) by a third independent reader whose conclusion was decisive. Positive microscopy results were recorded as the geometric means of the two reader’s results or the geometric means of the two geometrically closest reading in case of third reading. These results were expressed as asexual parasites per microlitre by using the patient’s white blood cell (WBC) count. A selection of slides (5%) was re-read by an independent expert microscopist for quality assurance.

#### Microbiology

For blood cultures, 1–3 ml of venous blood was collected into a paediatric blood culture bottle (BD BACTEC Peds Plus™/F, Becton–Dickinson and Company, Sparks, Maryland, USA) and incubated in a BACTEC 9050 instrument (Becton–Dickinson) for a total of 5 days according to the manufacturers’ protocol. If flagged for growth the cultures were Gram stained, sub-cultured on Eosin-Methylene Blue (EMB) agar, 5% Sheep Blood agar (bioMérieux, Marcy-l’Etoile, France) and chocolate + isoVitalex agar, and incubated at 35–37 °C for 24 h in atmospheric condition for EMB and at CO_2_ for Sheep Blood agar and chocolate + isoVitalex agar. Isolates were identified by standard microbiological methods like described in Mackie and McCartney Practical Medical Microbiology [18] and biochemical test (API strips, bioMerieux Marcy-l’Etoile, France).

Urine culture was done when a urine sample was positive for leucocytes and nitrite as indicated by a urine dipstick (Standard Diagnostics, UroColor, Inc, Korea). Urine was inoculated on CLED (Cystine Lactose Electrolyte Deficient) and EMB agar. The cultures were incubated at 37 °C during 24 h. Only samples that generated pure bacterial growth of more than 10^5^ colonies forming units (CFU)/ml were regarded as yielding significant bacteriuria. A count of ≤10^5^ CFU/ml was regarded as negative. Mixed growths (growth of more than one species in a sample, in particular growth of normal skin flora picked up during urine collection) were regarded as contaminated and therefore disregarded. A second urine sample was not collected. Pathogen identification was done by standard microbiology biochemical methods (API system, bioMerieux Marcy-l’Etoile, France).

Stool samples were specifically analysed for group A rotavirus using one step rotavirus and adenovirus serotype 40/41 in human feces test (SD Bioline Rota/Adeno; Standard Diagnostic, Inc., Korea) as these pathogens are considered to cause fever.

For the purpose of this study bacterial bloodstream infections (bBSI), viral gastro-intestinal infection (vGII) caused by rotavirus and adenovirus, and urinary tract infections (UTI) were considered as alternative cause of fever, different from malaria [19–22].

### Data analysis

Double entry of data was performed by two different persons by using OpenClinica software. The data analysis was done by using STATA 13. Description of qualitative and quantitative variables were performed by using proportion and mean or median, respectively. The geometric mean was used to express the parasite densities. The performance of RDT was evaluated compared to microscopy by calculating the sensitivity, the specificity, negative and positive predictive value of the RDT.

To assess the influence of alternative causes of fever as defined for the purpose of this study and prior use of anti-malarials on the RDT performance, it was assumed that infection status and prior anti-malarial use was known before RDT testing and, therefore, a correction was done by removing all confirmed cases of bloodstream infections, urinary tract infections, viral gastro-intestinal infections and prior use of anti-malarials within 2 weeks from the analysis either separately or together.

The rainy season was from July to November and dry season from December to June, and an analysis, done by season next to a whole year, was done on the results The Cohen’s Kappa coefficient was computed to assess to agreement between microscopy and RDT. p ≤ 0.05 was considered statistical significant.

## Results

### General study characteristics

In total 1447 children under-5 years of age attending the peripheral health facilities or the CMA Saint Camille of Nanoro between January to December 2015 were screened. Of these children, 684 children under-5 years with axillary temperature ≥37.5 °C at presentation were enrolled in the study. One child was excluded at a later stage because the malaria RDT was not performed at enrolment. The baseline characteristics of the remaining 683 febrile children are summarized in Table 1.

**Table 1:** Baseline characteristics of the study population enrolled at the health facilities and the district hospital

	Included N = 683	Malaria microscopy positive n = 340	Malaria microscopy negative n = 343	*Pf*HRP2 positive n = 475	*Pf*HRP2 negative n = 208
Sex (%)					
Male	369 (54.0)	176 (51.8)	193 (56.3)	256 (53.9)	113 (54.3)
Female	314 (46.0)	164 (48.2)	150 (43.7)	219 (46.1)	95 (45.7)
Age (%), months					
≤12	200 (29.3)	64 (18.8)	136 (39.7)	110 (23.2)	90 (43.3)
>12	483 (70.7)	276 (81.2)	207 (60.3)	365 (76.8)	118 (56.7)
Temperature (%), °C					
≥37.5–≤38.5	340 (49.8)	153 (45.0)	187 (54.5)	221 (46.5)	119 (57.2)
>38.5–≤39.5	241 (35.3)	123 (36.2)	118 (34.4)	177 (37.3)	64 (30.8)
>39.5	102 (14.9)	64 (18.8)	38 (11.1)	77 (16.2)	25 (12.0)
All causes (%)	73 (10.7)	11 (3.2)	62 (18.1)	47 (9.9)	26 (12.5)
BSI (%)	41 (6.0)	6 (1.8)	35 (10.2)	29 (6.1)	12 (5.8)
UTI (%)	10 (1.5)	3 (0.9)	7 (2.0)	9 (1.9)	1 (0.5)
Rotavirus/adenovirus (%)	25 (3.7)	3 (0.9)	22 (6.4)	12 (2.5)	12 (5.8)

BSI: bloodstream infection; UTI: urinary tract infection; *Pf*HRP2: *Plasmodium falciparum* histidine-rich protein-2

### Performance of RDT compared to microscopy and malaria treatment

The diagnostic performance of the malaria RDT-*Pf*HRP2 is reported in Table 2. *Plasmodium falciparum* infection was diagnosed by microscopy in 49.8% (340/683) and by RDT-*Pf*HRP2 in 69.5% (475/683) of the febrile children. The proportion of fever attributable to malaria as determined by expert microscopy was lower in the dry season (29.1%; 100/344) than in the rainy season (70.8%; 240/339). Out of the 475 febrile children with a positive RDT result, 29.7% (141/475) were found negative by expert malaria microscopy and thus considered RDT false positive. Stratifying for seasonality showed that RDT false positives were more prevalent during the dry season (46.4%; 84/181) compared to the rainy season (19.4%; 57/294). There were 2.9% (6/208) false negative cases; i.e. children positive by microscopy but negative by RDT.

**Table 2:** Agreement between expert malaria microscopy and *Pf*HRP2 RDT

	True positive n (%)	True negative n (%)	False positive n (%)	False negative n (%)
Whole year (N = 683)	334 (48.9)	202 (29.6)	141 (20.6)	6 (0.9)
Dry season (N = 344)	97 (28.2)	160 (46.5)	84 (24.4)	3 (0.9)
Rainy season (N = 339)	237 (69.9)	42 (12.4)	57 (16.8)	3 (0.9)

Number of observed agreements: 536 (78.48% of the of the observation)

Number of agreement expected by chance: 340.9 (49.91% of the observation)

Kappa = 0.570

SE of Kappa = 0.029

95% confidence interval: from 0.514 to 0.627

The strength of agreement is considered to be “moderate”

p < 0.00001

The sensitivity, specificity, positive predictive value (PPV) and negative predictive value (NPV) of the RDT-*Pf*HRP2 were 98.2% (334/340), 58.9% (202/343), 70.3% (334/475) and 97.1% (202/208), respectively (see Table 3). The agreement between microscopy and RDT was moderate with a Cohen’s Kappa coefficient of 0.57 (p < 0.00001). Interestingly the *Pf*HRP2 RDT showed to be more specific (65.6%; 160/244) in the dry season than during the rainy season (42.9%; 42/99) (see Table 3).

**Table 3:** Diagnostic performance of *Pf*HRP2-RDT compared with expert microscopy (gold standard) for detection of malaria in febrile children

Accuracy parameters	Value % (n/N)	Confidence intervals (95% CI)
Diagnostic performance all year		
Sensitivity	98.2 (334/340)	96.2–99.3
Specificity	58.9 (202/343)	53.5–64.1
Positive predictive value	70.3 (334/475)	66–74.4
Negative predictive value	97.1 (202/208)	93.8–98.9
Diagnostic performance during dry season		
Sensitivity	97.0 (97/100)	91.5–99.4
Specificity	65.6 (160/244)	59.2–71.5
Positive predictive value	53.6 (97/181)	46–61
Negative predictive value	98.2 (160/163)	94.7–99.6
Diagnostic performance during rainy season		
Sensitivity	98.8 (237/240)	96.4–99.7
Specificity	42.4 (42/99)	32.5–52.8
Positive predictive value	80.6 (237/294)	75.6–85
Negative predictive value	93.3 (42/45)	81.7–98.6

### Other causes of fever and influence of previous malaria treatment on RDT performance

In the RDT malaria false positive group, 25.5% (36/141) of the children were found to have other causes of fever based on the laboratory findings (Table 4). The most common was a bacterial bloodstream infection (bBSI; 16.3%; 23/141), followed by rotavirus and adenovirus infections (vGII; 6.4%; 9/141) and urinary tract infection (UTI; 4.3%; 6/141). For the alternative causes of fever, 49.3% (36/73) were diagnosed in malaria RDT-*Pf*HRP2 false positive group, 35.6% (26/73) in malaria RDT-*Pf*HRP2 true negative and 15.1% (11/73) in malaria RDT-*Pf*HRP2 true positive group. The difference of alternative causes of fever between malaria RDT-PfHRP2 false positive group 25.53% (36/141) and malaria RDT-PfHRP2 true negative group 12.38% (26/202) was statistically significant (p = 0.002). It was also observed that there was no significant difference (p = 0.632) in geometric mean parasite densities between the true RDT malaria positive children either with (26,279.52: 21,1349.7–32,347.7) or without (23,957.4: 19,378.7–29,617.9) an additional cause of fever.

**Table 4:** Laboratory findings and previous antimalarial use reported per RDT outcome

Characteristics	n/N (%)
False malaria positive	141
Laboratory data	
Alternative cause of fever	36/141 (25.5)
Bacterial bloodstream infection (BSI)	23/141 (16.3)
Rotavirus/adenovirus	9/141 (6.4)
Urinary tract infection (UTI)	6/141 (4.3)
Previous antimalarial use reported	14/141 (9.9)
True malaria negative	202
Laboratory data	
Alternative cause of fever	26/202 (12.9)
Bloodstream infection (BSI)	12/202 (5.9)
Rotavirus/adenovirus	13/202 (6.4)
Urinary tract infection (UTI)	01/202 (0.5)
Previous antimalarial use reported	19/202 (9.4)
True malaria positive	334
Laboratory data	
Alternative cause of fever	11/334 (3.3)
Bloodstream infection (BSI)	06/334 (1.8)
Rotavirus/adenovirus	03/334 (0.9)
Urinary tract infection (UTI)	03/334 (0.9)
Previous antimalarial use reported	01/334 (0.3)

In the true (by microscopy) malaria negative group, 12.9% (26/202) of the children were infected by pathogens that are known to cause fever and that were assessed in this study. Of the children that were confirmed to be positive for malaria by microscopy, only 3.3% (11/334) were co-infected with other causes of fever that were considered in this study (Table 4). During the data collection, 5.0% (31/683) parents or guardians declared to have given anti-malarial medication to her/his child within 2 weeks before inclusion. Among these children there were 17 cases in the true malaria negative group and 13 cases in the false malaria positive group. One child in the true malaria positive group declared to have taken anti-malarial within 2 weeks before inclusion.

### Correction of RDT performance by using outcomes of other diagnostic testing

One of the aims of the study was to assess the sensitivity and specificity of the employed RDT if it would be possible to exclude patients with prior anti-malarial use and bacterial infections. The corrected performance of the RDT is reported in Table 5 and the corrected accuracy in Table 6. In that case, the overall specificity of the RDT would have increased from 58.9% (202/343) to 63.0% (162/257) and the PPV from 70.3% (334/475) to 77.1% (320/415) (Table 6). The sensitivity (98.2%; 334/340) would not change and the NPV would have decreased slightly from 97.1% (202/208) to 96.4%). The agreement after correction between the microscopy and the RDT was moderate with a Cohen’s Kappa coefficient of 0.62 (p < 0.001). This effect would be larger in the rainy season as the specificity would have increased from 42.4% (42/99) to 50.7% (35/69) and the PPV from 80.6% (237/294) to 87.2% (231/265). The effect would have been less marked in the dry season where the specificity would have increased from 65.6% (160/244) to 67.6% (127/188) and the PPV from 53.6% (97/181) to 59.3% (89/150). Taking the result of bBSI into account had the largest effect on RDT performance and prior anti-malarial intake had the smallest effect.

**Table 5:** Diagnostic performance of the RDT after correcting for the influence of other febrile illnesses and previous antimalarial intake per season

	True positive n (%)	True negative n (%)	False positive n (%)	False negative n (%)
Performance if bBSI, UTI, vGII and previous antimalarial intake are excluded				
Whole season (N = 583)	320 (54.9)	162 (27.8)	95 (16.3)	6 (1.0)
Dry season (N = 280)	89 (31.8)	127 (45.4)	61 (21.8)	3 (1.1)
Rainy season (N = 303)	231 (76.2)	35 (11.5)	34 (11.2)	3 (1.0)
Performance after exclusion of bBSI				
Whole season (N = 642)	328 (51.1)	190 (29.6)	118 (18.4)	6 (0.9)
Dry season (N = 326)	91 (27.9)	153 (46.9)	79 (24.2)	3 (0.9)
Rainy season (N = 316)	237 (75.0)	37 (11.7)	39 (12.3)	3 (0.9)
Performance after exclusion of UTI				
Whole season (N = 671)	329 (49.0)	201 (30.0)	135 (20.1)	6 (0.9)
Dry season (N = 342)	97 (28.4)	159 (46.5)	83 (24.3)	3 (0.9)
Rainy season (N = 329)	232 (70.5)	42 (12.8)	52 (15.8)	3 (0.9)
Performance after exclusion of vGII				
Whole season (N = 647)	326 (50.4)	188 (29.1)	127 (19.6)	6 (0.9)
Dry season (N = 318)	94 (29.6)	147 (46.2)	74 (23.3)	3 (0.9)
Rainy season (N = 329)	232 (70.5)	41 (12.5)	53 (16.1)	3 (0.9)
Performance if UTI and vGII are excluded				
Whole season (N = 615)	325 (52.8)	171 (27.8)	113 (18.4)	6 (1.0)
Dry season (N = 295)	94 (31.9)	133 (45.1)	65 (22.0)	3 (1.0)
Rainy season (N = 320)	231 (72.2)	38 (11.9)	48 (15.0)	3 (0.9)
Performance if antimalarial intake in the previous 2 weeks is excluded				
Whole season (N = 648)	333 (51.4)	182 (28.1)	127 (19.6)	6 (0.9)
Dry season (N = 318)	97 (30.5)	143 (45.0)	75 (23.6)	3 (0.9)
Rainy season (N = 330)	236 (71.5)	39 (11.8)	52 (15.8)	3 (0.9)

Number of observed agreements: 499 (81.80% of the of the observation)

Number of agreement expected by chance: 314.7 (51.59% of the observation)

Kappa = 0.624

SE of Kappa = 0.030

95% confidence interval: from 0.565 to 0.684

The strength of agreement is considered to be “good”

p < 0.00001

**Table 6:** Accuracy of RDT compared to expert microscopy for the detection of malaria in febrile children after exclusion of children with other bacterial or viral infections and previous antimalarial intake stratified per season

Accuracy parameters	Whole year		Dry season		Rainy season	
	Value % (n/N)	95% CI	Value % (n/N)	95% CI	Value % (n/N)	95% CI
Accuracy after exclusion of bBSI, UTI, vGII and previous antimalarial intake						
Sensitivity	98.2 (320/326)	96.0–99.3	96.7 (89/92)	90.8–99.3	98.7 (231/234)	96.3–99.7
Specificity	63.0 (162/257)	56.8–69.0	67.6 (127/188)	60.4–74.2	50.7 (35/69)	38.4–63
PPV	77.1 (320/415)	72.8–81.1	59.3 (89/150)	51–67.3	87.2 (231/265)	82.5–90.9
NPV	96.4 (162/168)	92.4–98.7	97.7 (127/130)	93.4–99.5	92.1 (35/38)	78.6–98.3
Accuracy after exclusion of bBSI						
Sensitivity	98.2 (328/334)	96.1–99.3	96.8 (91/94)	91–99.3	98.8 (237/240)	96.4–99.7
Specificity	61.7 (190/308)	56–67.1	65.9 (153/232)	59.5–72	48.7 (37/76)	37–60.4
PPV	73.5 (328/446)	69.2–77.6	53.5 (91/170)	45.7–61.2	85.9 (237/276)	81.2–89.8
NPV	96.9 (190/196)	93.5–98.9	98.1 (153/156)	94.5–99.6	92.5 (37/40)	79.6–98.4
Accuracy after exclusion of UTI						
Sensitivity	98.2 (329/335)	96.1–99.3	97.0 (97/100)	91.5–99.4	98.7 (232/235)	96.3–99.7
Specificity	59.8 (201/336)	54.4–65.1	65.7 (159/242)	59.4–71.7	44.7 (42/94)	34.4–55.3
PPV	70.9 (329/464)	66.5–75	53.9 (97/180)	46.3–61.3	81.7 (232/284)	76.7–86
NPV	97.1 (201/207)	93.8–98.9	98.1 (159/162)	94.7–99.6	93.3 (42/45)	81.7–98.6
Accuracy after exclusion of vGII						
Sensitivity	98.2 (326/332)	96.2–99.3	96.9 (94/97)	91.2–99.4	98.7 (232/235)	96.4–99.7
Specificity	59.7 (188/315)	53.3–64.3	66.5 (147/221)	59.8–72.5	43.6 (41/94)	31.9–52.2
PPV	72.0 (326/453)	67.1–75.6	55.9 (94/168)	47.8–63.2	81.4 (232/285)	75.6–85
NPV	96.9 (188/194)	93.4–98.9	98.0 (147/150)	94.3–99.6	93.2 (41/44)	81.3–98.6
Accuracy after exclusion of UTI and vGII						
Sensitivity	98.2 (325/331)	96.1–99.3	96.9 (94/97)	91.2–99.4	98.7 (231/234)	96.3–99.7
Specificity	60.2 (171/284)	54.3–65.9	67.2 (133/198)	60.2–73.7	44.2 (38/86)	33.5–55.3
PPV	74.2 (325/438)	69.8–78.2	59.1 (94/159)	51.1–66.8	82.8 (231/279)	77.8–87
NPV	96.6 (171/177)	92.8–98.7	97.8 (133/136)	93.7–99.5	92.7 (38/41)	80.1–98.5
Accuracy after exclusion of antimalarial intake during the previous 2 weeks						
Sensitivity	98.2 (333/339)	96.2–99.3	97.0 (97/100)	91.5–99.4	98.7 (236/239)	96.4–99.7
Specificity	58.9 (182/309)	53.2–64.4	65.6 (143/218)	58.9–71.9	42.9 (39/91)	32.5–53.7
PPV	72.4 (333/460)	68.1–76.4	56.4 (97/172)	48.6–63.9	81.9 (236/288)	77–86.2
NPV	96.8 (182/188)	93.2–98.8	97.9 (143/146)	94.1–99.6	92.9 (39/42)	80.5–98.5

PPV: positive predictive value; NPV: negative predictive value

## Discussion

This study showed that a large proportion of children treated for malaria based on a positive HRP2 RDT results were children who were not infected with malaria if the result of expert microscopy is considered as gold standard. Around 25% of these children actually had another treatable bacterial infection that is missed if only the result of the HRP2 RDT for malaria was followed for treatment. Adding a test for bacterial infections and the history of previous malaria treatment somewhat increased RDT test performance in terms of specificity and PPV. This may subsequently reduce malaria overtreatment. Interestingly seasonality had an influence on RDT performance as well as the contribution of other diseases that were assessed in this study on RDT performance. Possibly, the intensity of malaria transmission, which is different between the dry and the rainy season in the study area (Nanoro), could explain the influence of the seasonality on the performance of the RDT-*Pf*HRP2 as also suggested [14].

The most important finding of the present study is thus the relative high number of malaria RDT false positive results found, if expert malaria microscopy is considered as golden standard. The number found in the present study is in line with those reported by Maltha et al. and Tinto et al. in the same study area [15, 23] and several studies in other malaria endemic areas [24, 25]. In particular, it is noted that Malta et al. reported that 27% of the positive *Pf*HRP2 cases did not have an actual malaria infection and that *Pf*HRP2 is less specific during rainy season compared to dry season, which is in line with the study of Bisoffi et al. [14] and confirmed in the present study.

One of the generally accepted reasons for false positive RDT results is persisting antigen after adequate treatment or spontaneous remission [11]. However, in the present study the contribution of previous treatment seems to be little, as only 5% of the parents have reported previous anti-malarial treatment. Other factors that could have contributed to the observed false positivity of the RDT can be as follows. Firstly, the sensitivity of microscopy may be below that of RDTs. It has for example been shown by Bisoffi et al. [14] that the RDT employed in that study had a LoD of 50 parasites/µl and that some of the false positive RDT results are in fact false negative microscopy results. However, the microscopists in the current study are submitted to an external control programme and only accredited microscopists are allowed to read malaria thick blood smears [21]. The LoD of this expert microscopy is around 10 parasites/μl and of the employed RTD (recommended by the NMCP of Burkina Faso) is also around 50 parasites/μl as reported by Tinto et al. [21]. The lowest parasite density observed in our study was 72 parasites/μl. Therefore it is not very likely that false negative microscopy has attributed much to false positive RDTs observed in the present study. Future studies should take this point into consideration and may want to establish the LoD of the employed RDT in advance.

Secondly, the RDT-*Pf*HRP2 used in the present study was ordered in the framework of the implemented programme for the management of malaria in the health district. The research team did not have any control over the transportation and storage conditions of the tests and there is a certain level of uncertainty if these might have affected test performance. Albertini et al. [26] showed that transport and storage conditions of malaria RDTs often exceed recommended temperatures and this may affect test performance. However, the tests were always handled according to the NMCP guidelines in place and expired tests were never used. Thirdly, the study nurses were not specifically trained in performing the malaria RDT for the purpose of the study. They performed the test according to their routine practice. Possibly a refresher training would have been appropriate. Whether this explains the higher number of false positives is questionable as health workers sometimes tend to ignore a weak positive line (thus scoring the test as “negative”) rather than considering a negative test as being positive [23–25].

It should be noted that this study was designed to assess the performance of the RDT under actual field conditions and therefore the mentioned limitations are in fact a reflection of the real-life situation. The observation remains that the overestimation of malaria infections by *Pf*HRP2 is a real concern as found in this study and also reported in several studies [15, 23–25] and needs to be taken into consideration in the management of febrile cases, in particular not to overlook the real cause of fever.

The justification to choose for the implementation of a malaria RDT-*Pf*HRP2 in many malaria endemic countries is based on test performance as reported by WHO panel studies for this RDT [17].The WHO recommends selecting a RDT based on its sensitivity and specificity (which both should be ≥95% [27]). The present study only focused on the use of one particular brand of RDT, which might be seen as a limitation of the current study. However, the employed RDT is implemented by the NMCP of Burkina Faso and, therefore, a diagnostic evaluation is warranted. The employed RDT has a very high sensitivity and NPV as confirmed in the present study. These characteristics enables the identification of the majority of malaria positive cases by RDT (98.24%) and these cases have been treated accordingly, in line with WHO and NMCP guidelines. In addition, its excellent NPV ensures that an important part of the true malaria negative cases (97.12%) are correctly diagnosed as negative by RDT-*Pf*HRP2 and it emphasizes the need to further assess the true aetiology of the (non-malaria) fever in the sick child with a specific attention to other infections. In the routine practice of CRUN, patients will first be asked for symptoms and submitted to clinical examination prior to doing a RDT and obviously if they have clear symptoms of pneumonia or urinary or gastro-intestinal infections these will be treated too even if a RDT is positive for malaria.

It is important to note that the low specificity and PPV found in this study can negatively affect the clinical management of febrile cases. Almost 30% of the malaria RDT-*Pf*HRP2 positives cases were in fact malaria negative, as determined by microscopy, and these children received, in line with the NMCP and WHO malaria treatment guidelines, malaria treatment as their test was positive, but unnecessary (hence over prescription of anti-malarials), as they actually do not have the disease [28] and most likely should have been treated with appropriate antibiotics as an alternative to treat the cause of fever.

Moreover, the poor PPV resulted in the misdiagnosis of the actual cause of febrile illness in around 30% of the children that were tested positive by RDT testing, but actually had another disease that caused their fever. Importantly, almost 50% of the alternative cause of fevers found in this study were actually diagnosed in the children that had false positive results with the RDT-*Pf*HRP2. This could leave clinicians and health workers with a huge dilemma in the case of a malaria positive RDT, i.e. either providing immediately anti-malarials or first further investigate other possible causes of fever. In a recent study a proposition of performing a two-step RDT with *Pf*HRP2 and *Plasmodium* Lactate Dehydrogenase (pLDH) was made to improve the specificity *P. falciparum* diagnosis in high transmission settings with little loss of sensitivity [29]. HRP2 positive/pLDH negative cases where further studied by microscopy as a confirmative test. Therefore, it is now proposed that additional testing with a urine dipstick for UTI and a faeces dipstick for vGII can further strengthen this algorithm and will further increase the diagnostic performance and hence the management of febrile cases. A prospective study to further validate such an algorithm in routine practice is needed.

It is also essential to emphasize that clinicians and health workers must be aware that if after anti-malaria treatment with adequate drugs the fever does not subside alternative causes of fever must be considered. In the present study it was found that 3.29% of the children that had a confirmed malaria infection, also had another infection that causes fever and these also need to be adequately treated.

Prior anti-malarial treatment mainly concerned principally RDT false positive (and thus microscopy negative) and true negative cases. This finding shows that the anti-malarials prescribed in the study region (principally artemisinin-based combination therapy for uncomplicated malaria) are highly efficient. Therefore, RDT positive cases with prior anti-malarial treatment (<3 weeks) are likely to be false positive, and clinicians and health works should thus be aware of this phenomenon and always thoroughly ask parents or guardians about previous intake use of drugs [4].

The risk of having incorrect results by microscopy is in the present study very low. The microscopists who took part in the present study are submitted to regular certification programmes to ensure the quality of the malaria slide reading performed. Therefore, the persistence of HRP2 antigens detectable up to 3 weeks after successful anti-malarial treatment is considered as an important cause of the false positives [4].

The present study found only few false negative cases (2.88%; 6/208); i.e. children positive by microscopy but negative by RDT. There is increasing concern that *P. falciparum* parasites having deletion(s) in the HRP2 gene yield false negative RDT results [30, 31]. This phenomenon has not been studied in Nanaro, but will require attention in future studies as HRP2-based tests are recommended by the NMCP.

## Conclusions

In conclusion, RDTs based on *Pf*HRP2 have a good sensitivity and negative predictive value, but their use in malaria endemic areas may result in missing true causes of fever mainly in the malaria false positive group. This puts a dilemma to clinicians and health workers that could be circumvented by using a second malaria RDT based on a different target, such as pLDH and/or using other simple diagnostic tests like urine and feces dipstick. In addition, the development of practical tool to screen the other causes of fever in malaria endemic area, should contribute to save live, but also to improve the performance of the malaria RDT view the few cases of co-infection of malaria with these other causes of fever.

## Abbreviations

bBSI: bacterial bloodstream infections
CLED: cystine lactose electrolyte deficient
CFU: colonies forming units
CMA: Centre Medical avec Antenne Churigicale
CRUN: Clinical Research Unit of Nanoro
EMB: Eosin-Methylene Blue
NMCP: National Malaria Control Programme
NPV: negative predictive value
*Pf*HRP2: *Plasmodium falciparum* histidine-rich protein-2
RDT-*Pf*HRP2: *Pf*HRP2-based RDT
PCR: polymerase chain reaction
PPV: positive predictive value
RDT: rapid diagnostic test
SD: standard diagnostic
UTI: urinary tract infections
vGII: viral gastro-intestinal infection
WBC: white blood cell
WHO: World Health Organization
